# Segmental bioimpedance and anthropometry improve machine learning prediction of grip strength in healthy young adults

**DOI:** 10.3389/fbioe.2026.1736894

**Published:** 2026-01-22

**Authors:** Helen Najjar, Khouloud Issa, Heba M. Badawe, Massoud L. Khraiche

**Affiliations:** Neural Engineering and Nanobiosensors group, Biomedical Engineering Program, Maroun Semaan Faculty of Engineering and Architecture, American University of Beirut, Beirut, Lebanon

**Keywords:** anthropometry, bioimpedance, hand grip strength, machine learning, wearables

## Abstract

**Objective:**

Accurate, non-invasive assessment of muscle strength remains a key challenge for functional health monitoring and wearable systems. This study investigates whether segmental bioimpedance (BioZ) and anthropometry measurements from the wrist and forearm can predict hand grip strength (HGS) in healthy young adults, and characterizes how measurement site, frequency, and applied pressure influence BioZ signal behavior, which are critical factors for translating BioZ into wearable applications.

**Methods:**

We recruited twenty healthy young adults who underwent standardized HGS testing alongside segmental BioZ measurements at the wrist and forearm using a bipolar electrode configuration. Anthropometric variables including age, height, body mass, and limb circumference were recorded. Nonparametric statistical analyses were used to examine anatomical site-specific differences and associations among BioZ, circumference, and HGS. Multiple linear regression (MLR) and random forest (RF) regression models were developed to estimate HGS from anthropometric and localized BioZ features and evaluated using leave-one-out cross validation. In addition, exploratory single-subject experiments were conducted to assess BioZ responses to varying frequency, applied pressure, and electrode configuration at both anatomical sites.

**Results:**

At the cohort level, forearm BioZ values were higher than wrist BioZ (p = 0.0001) and inversely correlated with forearm circumference (ρ = −0.54, p = 0.014). Forearm circumference showed the strongest positive association with HGS, while forearm BioZ exhibited a moderate inverse association. Incorporating localized forearm BioZ into baseline improved predictive performance (RF regression: R^2^_cv_ = 0.44). A size-normalized BioZ index further enhanced prediction accuracy, achieving the highest in RF regression models (R^2^_cv_ = 0.56). Frequency- and pressure-dependent analyses revealed that high-frequency BioZ increased linearly with applied pressure, whereas low-frequency BioZ exhibited non-linear and less stable behavior, suggesting sensitivity to tissue compression and local fluid redistribution.

**Conclusion:**

This pilot study demonstrates that localized, size-normalized forearm BioZ provides physiologically complementary information to basic anthropometry for estimating HGS in healthy young adults. By integrating cohort-level modeling with exploratory mechanistic experiments, the findings provide insight into the anatomical and mechanical determinants of localized BioZ behavior. This supports the potential utility of combining experimental and computational approaches to inform the future development of next-generation BioZ-based wearable systems for non-invasive assessment of muscle strength, rehabilitation progress, and early signs of muscular decline.

## 1 Introduction

Wearable health technologies are increasingly shaping how we monitor fitness, recovery, and chronic disease risk, yet most devices still rely on indirect measures, such as heart rate or step counts. To advance beyond basic activity tracking, there is growing interest in integrating biomarkers that more directly reflect muscle strength and tissue composition (Daniore et al., 2024). Hand grip strength (HGS) is a simple, non-invasive biomarker that reflects functional capacity and overall health status. Beyond its role in everyday activities, HGS has been consistently linked to chronic disease risk, sarcopenia, cardiovascular and cancer-related mortality, and even cognitive decline (Ho et al., 2019; Vaishya et al., 2024; da Silva et al., 2018). Lower HGS is also associated with poor mental health outcomes including depression, and a reduced quality of life (Fielding et al., 2011; Kwak and Kim, 2019). HGS reflects activity of both extrinsic forearm flexors and intrinsic hand muscles, coordinated with wrist stabilizers. For this reason, HGS is considered a global marker of neuromuscular health, as it integrates signals from multiple muscle groups and the nervous system.

Beyond functional measures such as HGS, segmental bioimpedance (BioZ) analysis characterizes tissue composition, muscle status and fluid balance (Puertas et al., 2021; Nescolarde et al., 2013). BioZ is a non-invasive, safe and versatile technique in which a small alternating current is applied across body tissues to measure electrical impedance and derive physiological parameters (Naranjo-Hernández et al., 2019; Borga et al., 2018; Amini et al., 2018; Campa et al., 2021). A key advantage of BioZ is its ability to deliver real-time, continuous monitoring of cardiovascular and fluid status, enabling early detection of changes such as fluid overload, edema, and cardiac dysfunction (Naranjo-Hernández et al., 2019; Kazory, 2017). This approach has been used to detect sarcopenia, monitor recovery, and evaluate post-surgical swelling, often outperforming conventional anthropometric methods (Konečná et al., 2023; Pichonnaz et al., 2015). Measurement accuracy, however, is influenced by electrode placement and type (Vaishya et al., 2024; Kwak and Kim, 2019; Noh and Park, 2020), skin contact quality (Yang et al., 2022), excitation frequency, and applied pressure (Taji et al., 2018). These factors are critical for translating BioZ into practical wearable systems. Multi-frequency BioZ further enhances diagnostic potential by distinguishing between extracellular and intracellular fluid compartments and assessing cell integrity. Specifically, low-frequency signals (<50 kHz) are limited to extracellular pathways due to cell membrane impedance, while high-frequency signals (>200 kHz) can penetrate cell membranes, enabling assessment of intracellular fluid and cellular integrity (Konečná et al., 2023; Pichonnaz et al., 2015). Recent advances in dry electrodes and strap-based systems highlight the potential for gel-free, continuous monitoring in wearables devices, opening pathways for its broader clinical and consumer applications (Escobar-Linero et al., 2023). Machine learning (ML)-based signal correction complements these advances by enhancing measurement reliability (Nazarzadeh et al., 2024; Zhou et al., 2023; Al-Nabulsi et al., 2024; Lindholm et al., 2018). Despite their diagnostic potential, both HGS and BioZ remain underutilized in routine healthcare and are largely absent from wearable devices. This underuse stems from several challenges, including variability in measurement protocols, lack of clinical standardization, technical barriers for wearable integration, and limited clinician familiarity (Nazarzadeh et al., 2024; Zhou et al., 2023; Al-Nabulsi et al., 2024; Lindholm et al., 2018). Although HGS provides a direct functional measure of muscular strength and BioZ captures underlying physiological and tissue-level properties, their combined integration remains largely under-investigated. To date, few studies have systematically assessed the complementary predictive value of combining HGS and BioZ within ML frameworks, leaving a gap in understanding how these modalities could enhance individualized health monitoring and risk prediction. In the Middle East and North Africa (MENA) region, recent studies have characterized HGS and body composition using BioZ, linking these measures to obesity, sarcopenia and frailty in older adults (Saadeddine et al., 2021; Kreidieh et al., 2020; al-Lababidi et al., 2025). However, data integrating BioZ, HGS, and ML in healthy young adults remain limited. By focusing on this population, the present pilot study establishes a reference framework that can support future comparisons across age groups, clinical conditions, and disease states.

In this work, we present the first integration of segmental BioZ from the wrist and forearm with anthropometry and HGS to predict muscle strength in healthy young adults from the MENA region. By benchmarking multiple linear regression (MLR) against random forest (RF) models, we show that combining localized electrical properties with simple anthropometric measures enhances the prediction of HGS beyond conventional approaches. We further investigate how measurement site and limb geometry influence BioZ behavior, while motivating future investigations into the effects of applied pressure and frequency, generating critical insights for both physiological interpretation and translation into practical wearable systems.

## 2 Materials and methods

### 2.1 Hand grip strength and bioimpedance study

#### 2.1.1 Participants

Twenty healthy young adults between the ages of 21 and 30 were recruited from the American University of Beirut (AUB) in Lebanon. This study was approved by the AUB Institutional Review Board (IRB) and conducted in accordance with the ethical standards set forth by the IRB (IRB ID: BIO-2024–0243). Participants reported no underlying conditions or recent surgeries affecting upper limb performance. All measurements were performed on the same arm, which corresponded to the participant’s self-reported dominant hand. BioZ recordings were taken at two anatomical sites, the wrist and forearm, and were later analyzed in relation to anthropometric measures and HGS performance. The sample size (n = 20) was based on feasibility and aligned with similar exploratory studies in wearable bioimpedance and strength assessment.

#### 2.1.2 Experimental setting and pre-test instructions

Testing was conducted in a quiet, temperature-controlled laboratory maintained at 22 °C–24 °C to minimize the influence of environmental factors such as sweating, elevated heart rate, agitation, and distraction. Participants were seated comfortably and allowed to rest for 5–10 min before testing to ensure a relaxed physiological state. To reduce physiological variability and improve measurement consistency, participants were instructed to abstain from food, caffeine, and smoking before testing, and to avoid vigorous physical activity on the day of testing to mitigate acute fluid-related fluctuations in BioZ (Lukaski et al., 1985; Kyle et al., 2004).

Prior to data collection, participants were introduced to the equipment and experimental procedures. A familiarization session was conducted to ensure all participants understood the required posture, placement, and gripping technique to mitigate variability and bias as best as possible. All equipment was sanitized before and after each session for hygiene, and the skin at the wrist and forearm electrode sites were cleaned using 70% isopropyl alcohol to reduce skin-electrode impedance and enhance signal quality. All measurements were conducted with participants seated upright in a standard chair, with their back supported and feet flat on the floor.

#### 2.1.3 Segmental bioimpedance data acquisition

BioZ data were collected using the MAX30001EVSYS (Maxim Integrated, CA, United States), a compact analog front-end (AFE) system capable of single-channel impedance monitoring. The device was configured to deliver an 8 µA sinusoidal current at 128 kHz through a bipolar electrode configuration, where the same two electrodes served as both current injection and voltage sensing terminals.

**FIGURE 1:**
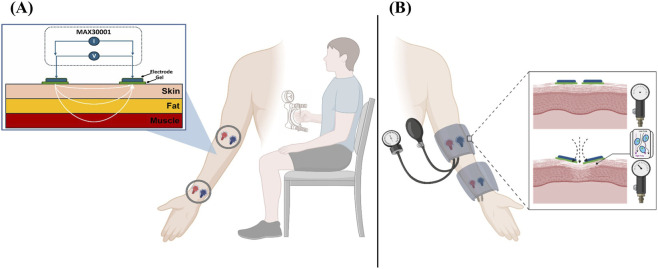
Experimental setup and participant positioning for **(A)** the hand grip strength (HGS) and bioimpedance (BioZ) study and **(B) **the investigation of BioZ parameters including frequency, pressure, and location.

BioZ measurements were recorded using standard wet Ag/AgCl electrodes at two anatomical locations on the volar (anterior) side of the arm: the wrist and the upper forearm as shown in Figure 1A. At the wrist, electrodes were placed approximately 2 cm proximal to the radiocarpal joint, centered over the flexor surface. At the forearm, electrodes were positioned 2 cm distal to the midpoint between the medial epicondyle of the humerus and the ulnar styloid process, aligned along the soft tissue of the inner forearm. Placement was carefully standardized across all participants to ensure consistent measurements. Each electrode had a contact area of 2.25 cm^2^ and was positioned longitudinally with a fixed center-to-center inter-electrode spacing of 2 cm, as shown in Figure 1A. A two-electrode configuration was used to reflect practical constraints relevant to wearable applications, including ease of integration, skin contact, and user comfort.

All BioZ recordings were conducted during the resting state prior to gripping. No muscle contraction or gripping activity was performed during these recordings to ensure that measurements reflected present resting tissue impedance and not transient changes associated with muscle activation. At each site (wrist and forearm), three BioZ measurements were obtained, with a 2-min rest between trials. The mean BioZ magnitude across the three trials at each anatomical location was calculated and used in subsequent analysis. A 3-min rest period was maintained when transitioning between anatomical sites. After each set of recordings, electrodes were carefully removed, and the skin was cleaned before data collection.

#### 2.1.4 Hand grip strength testing procedure

Isometric HGS was measured using a digital hydraulic hand dynamometer (FEI, White Plains, NY 10602, United States) as it is a standard method for measuring HGS. Participants were instructed to hold the dynamometer in their dominant hand while maintaining a seated posture without elevating or extending their arm forward as shown in Figure 1A. Following standardized HGS testing protocols, the elbow was flexed at 90°, the shoulder in a neutral position, and the wrist in a neutral (0° dorsiflexion) position. Participants were instructed to perform three maximal voluntary contractions, each lasting approximately 5 seconds, guided by the verbal cue: “I want you to squeeze as hard as you can until I say stop” (Vaishya et al., 2024). A 2-min rest interval was provided between successive trials to prevent localized fatigue and ensure recovery.

#### 2.1.5 Anthropometric measurements

To account for inter-individual differences in limb geometry, wrist and forearm circumference measurements were taken once, using a flexible, non-elastic tape. The wrist circumference was measured at the level of the radial and ulnar styloid processes, and the forearm circumference was measured at the widest region between the elbow and wrist. Participants also provided self-reported demographic and health-related data, including sex, age, height, body mass, and hand dominance.

#### 2.1.6 Statistical analysis and machine learning

Data processing and analysis were performed using IBM SPSS Statistics (version 31.0.1.0) and MATLAB (MathWorks, United States, 2024b), unless otherwise specified. A two-tailed significance level of p = 0.05 was adopted for inferential statistical analyses. Given the modest sample size (n = 20) and non-normal distributions of several variables, nonparametric inferentiel methods were employed. Wilcoxon signed-rank tests were used to compare wrist and forearm measurements for BioZ and circumference. Spearman correlation analyses were conducted to examine associations between (1) localized BioZ and circumference at each anatomical site and (2) localized BioZ and circumference measures and HGS. Sex-stratified descriptive statistics were reported for anthropometric variables but were not interpreted inferentially due to the imbalance between men and women.

Two supervised learning algorithms, specifically MLR and RF regression models, were used to model HGS and compare across progressively feature sets derived from anthropometric and segmental BioZ measurements. A baseline model including height and body mass served as a reference to assess the incremental contribution of anthropometric and localized BioZ measurements. Given the sample size (n = 20), model performance was evaluated using leave-one-out cross-validation (LOOCV) and quantified using cross-validated mean squared error (MSE), prediction error (RMSE), and the coefficient of determination (R^2^_cv_). Other models incorporated localized forearm BioZ and forearm circumference based on exploratory statistical findings. For RF regression, the minimum leaf size was set to 5 based on preliminary sensitivity analyses to balance bias and variance in small-sample settings.

### 2.2 Investigation of bioimpedance parameters: pressure, frequency and location

To establish how key measurement parameters such as pressure, frequency, and measurement site, affect tissue impedance in wearable BioZ applications, we conducted controlled measurements under varying conditions.

#### 2.2.1 Instrumentation and measurement setup

Experiments were conducted using the CORELAB EIT (Instruments, United States) to record all impedance measurements (128 kHz). All recordings were performed using dry Ag/AgCl electrodes in the same distance as mentioned in the above protocol. The forearm and wrist were selected as the two anatomical sites for BioZ evaluation, as shown in Figure 1B.

These experiments were conducted on a single participant to investigate how anatomical location, pressure magnitude, and measurement frequency influence impedance characteristics. The goal of this part of the study was to investigate various factors that affect BioZ acquisition, particularly in the context of wearable sensor design. Signal processing and data analysis were performed using MATLAB (MathWorks, United States) and GraphPad Prism (Boston, United States). Impedance behavior was compared across different conditions with significance determined at p = 0.05.

#### 2.2.2 Forearm and wrist bioimpedance measurements

At the forearm, five pressure levels were applied using the cuff system mentioned above. The forearm tolerated higher pressures levels than the wrist, permitting a broader application range without discomfort. Impedance was measured at 1 MHz to assess high frequency behavior and 500 Hz to capture low-frequency responses. Additionally, continuous BioZ recordings were conducted at 1 MHz over 200 s to monitor real-time impedance changes as pressure was gradually applied and released to observe decompression effects and recovery dynamics.

For the wrist, electrodes were applied in two configurations: the Dorsal-Dorsal (DD) configuration, where both electrodes were placed on the dorsal surface of the wrist, and the Dorsal-Volar (DV) configuration, where one electrode was placed on the dorsal side and the other on the volar (anterior) side. Pilot testing showed that 100 mmHg caused pain at this site, whereas 90 mmHg was tolerable. Accordingly, wrist measurements were obtained at four pressure points. Each pressure level was held for 2 minutes during data acquisition, followed by a 10-min rest period to allow for complete tissue recovery, re-equilibration of interstitial fluid, and restoration of baseline impedance values (Dodde et al., 2012; Namkoong et al., 2024). These measurements were used to investigate frequency-dependent responses across various frequency bands. Pressure levels at both sites are grouped as baseline, low (30–40 mmHg), medium (60–80 mmHg) and high (90–100 mmHg).

## 3 Results

### 3.1 Bioimpedance, hand grip strength, and circumference

The final study population included 20 healthy Lebanese young adults (14 women, 6 men). The mean age was 24.5 ± 2.9 years. Overall anthropometric, BioZ, and HGS characteristics of the cohort are summarized in Table 1, along the total number of participants, the proportions of men and women, their mean and standard deviation (SD). Mean height and body mass were 163.5 ± 5.6 cm and 61.1 ± 9.0 kg for women (n = 14), and 175.2 ± 6.5 cm and 82.4 ± 11.4 kg for men (n = 6), respectively. All participants successfully completed the full set of measurements without complications.

**TABLE 1:** Characteristics of the pilot study population.

*N*	20
Gender	
Men (n)	6 (30%)
Women (n)	14 (70%)
Height (cm)	167 ± 7.93
Body Mass (kg)	67.48 ± 13.81
BMI (kg/m^2^)	24.01 ± 3.74
Age	24.50 ± 2.88
Wrist circumference (cm)	16.20 ± 1.47
Forearm circumference (cm)	24.89 ± 2.58
Forearm BioZ (Ω)	10.81 ± 2.69
Wrist BioZ (Ω)	7.38 ± 1.22
HGS (kg)	30.35 ± 10.32

The characteristics of this pilot study population variables were represented mean ± standard deviation (SD). Average HGS (kg) was calculated as the mean of six HGS trials.

**FIGURE 2:**
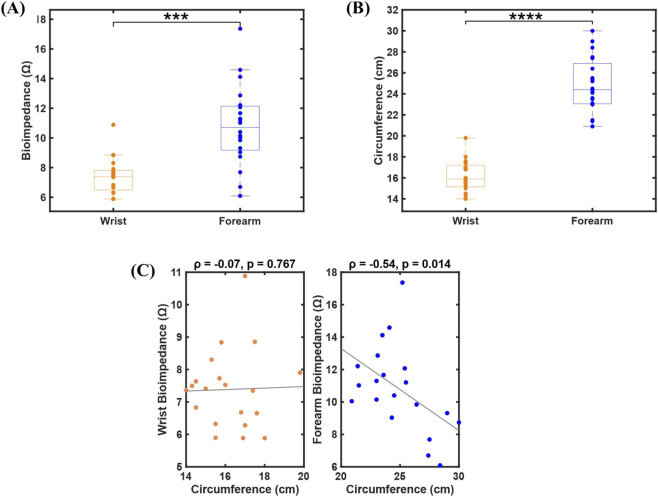
Comparison of wrist and forearm bioimpedance (BioZ) and circumference measurements. **(A)** BioZ (Ω) measured at the wrist and forearm showed a significant difference (Wilcoxon signed-rank test, p = 0.0001). **(B)** Circumference (cm) at the forearm was higher compared to the wrist (p = 0.00008). **(C)** Correlation between BioZ and circumference at both anatomical sites, with Spearman correlation coefficients and corresponding p-values indicated.

This cohort enabled exploratory analyses of muscle strength and localized BioZ using HGS and segmental BioZ measurements. BioZ and circumference measurements differed between the wrist and forearm across participants (Figure 2). Forearm BioZ values (mean ± SD: 10.8 ± 2.7 Ω) were higher than wrist BioZ values (mean ± SD: 7.4 ± 1.2 Ω), with a wider range observed at the forearm (6.1–17.4 Ω) compared to the wrist (5.9–10.9 Ω) (Wilcoxon signed-rank test, p = 0.0001; Figure 2A). The wider spread in forearm BioZ reflects greater inter-individual variability at that site. Similarly, forearm circumference (24.9 ± 2.6 cm) exceeded wrist circumference (16.2 ± 1.5 cm) across participants (p = 0.00008; Figure 2B). An inverse association was observed between forearm BioZ and forearm circumference (Spearman ρ = −0.54, p = 0.014; Figure 2C), indicating lower impedance values with increasing limb size. In contrast, no meaningful association was observed between wrist BioZ and wrist circumference.

**FIGURE 3:**
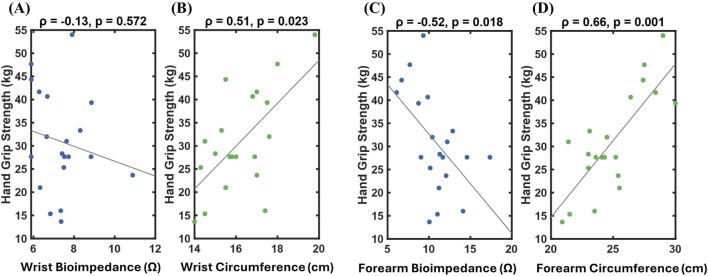
Correlation between hand grip strength (HGS) and local features: **(A)** wrist bioimpedance (BioZ), **(B)** wrist circumference, **(C)** forearm BioZ, and **(D)** forearm circumference, with Spearman correlation coefficients and corresponding p-values indicated.

To examine the relationships between localized limb measurements and muscular strength, associations between HGS and circumference and BioZ were evaluated at the wrist and forearm (Figure 3). Wrist BioZ showed no meaningful association with HGS (Spearman ρ = −0.13, p = 0.572; Figure 3A), while wrist circumference demonstrated a weak positive association (Spearman ρ = −0.51, p = 0.023; Figure 3B). At the forearm, BioZ showed a moderate inverse association with HGS (ρ = −0.52, p = 0.018; Figure 3C), whereas forearm circumference exhibited the strongest positive association with HGS (Spearman ρ = 0.66, p = 0.001; Figure 3D) forearm HGS. These associations were used to guide the selection of candidate features for subsequent predictive modeling.

### 3.2 Machine learning analysis

**FIGURE 4:**
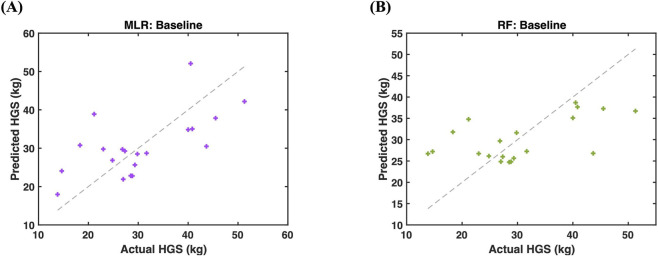
Regression modeling of hand grip strength (HGS) using baseline features (height and body mass) with **(A)** a multiple linear regression (MLR; purple) achieved R^2^_cv_ = 0.38 and RMSE_cv_ = 7.95 and **(B)** random forest regression (RF; green) achieved R^2^_cv_ = 0.32 and RMSE_cv_ = 8.29.

**FIGURE 5:**
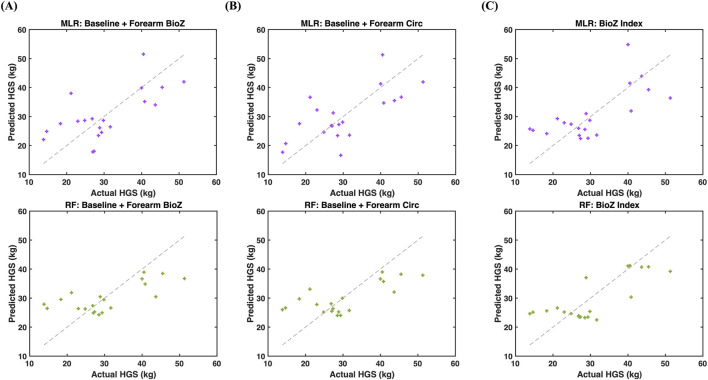
Regression modeling of hand grip strength (HGS) with added anthropometric and bioimpedance (BioZ) features at the forearm. **(A)** Models with baseline + forearm BioZ (top for MLR: R^2^_cv_ = 0.40 and RMSE_cv_ = 7.77 and bottom for RF regression: R^2^_cv_ = 0.41 and RMSE_cv_ = 7.73). **(B)** Models with baseline + Forearm circumference combined at the forearm (top for MLR: R^2^_cv_ = 0.45 and RMSE_cv_ = 7.49) and bottom for RF regression: R^2^_cv_ = 0.47 and RMSE_cv_ = 7.33). **(C)** Models with BioZ index only (top for MLR: R^2^_cv_ = 0.45 and RMSE_cv_ = 7.44 and bottom for RF regression: R^2^_cv_ = 0.56 and RMSE_cv_ = 6.68).

Predictive modeling results are shown in Figures 4, 5 and summarized in Table 2. Model performance was evaluated using LOOCV to account for the limited sample size (n = 20). Baseline models including height and body mass demonstrated modest predictive capability for HGS, with MLR and RF achieving R^2^_cv_ values of 0.38 and 0.32, respectively (Figure 5).

**TABLE 2:** Cross-validated performance of models for predicting HGS.

Model	Algorithm	MSE_cv_	RMSE_cv_	R^2^_cv_
Baseline	MLR	63.25	7.95	0.38
	RF	68.66	8.29	0.32
Baseline + forearm BioZ	MLR	60.42	7.77	0.40
	RF	56.92	7.73	0.41
Baseline + forearm circumference	MLR	56.15	7.49	0.45
	RF	53.34	7.33	0.47
Baseline + BioZ index	MLR	55.40	7.44	0.45
	RF	46.51	6.68	0.56

Incorporating segmental forearm BioZ into the baseline model improved predictive performance for both regression approaches, yielding R^2^_cv_ values of 0.40 for MLR and 0.41 for RF (Figure 5A). Further, the inclusion of forearm circumference resulted in additional improvements with R^2^_cv_ values of 0.45 for MLR and 0.47 for RF (Figure 5B).

Given the strong dependence of BioZ on limb geometry, a size-normalized forearm BioZ index was derived to reduce geometric confounding, as defined in Equation 1. Models incorporating this BioZ index achieved comparable performance to baseline anthropometric models with forearm circumference in linear regression (MLR R^2^_cv_ = 0.45) and superior predictive performance when modeled using RF regression (R^2^_cv_ = 0.56), with nonlinear models offering additional benefit only after geometric normalization of BioZ features (Figure 5C).

$$Forearm\;BioZ\;Index=\frac{{Circumference}^{2}}{BioZ}$$ (1)

### 3.3 Bioimpedance measurement parameters

**FIGURE 6:**
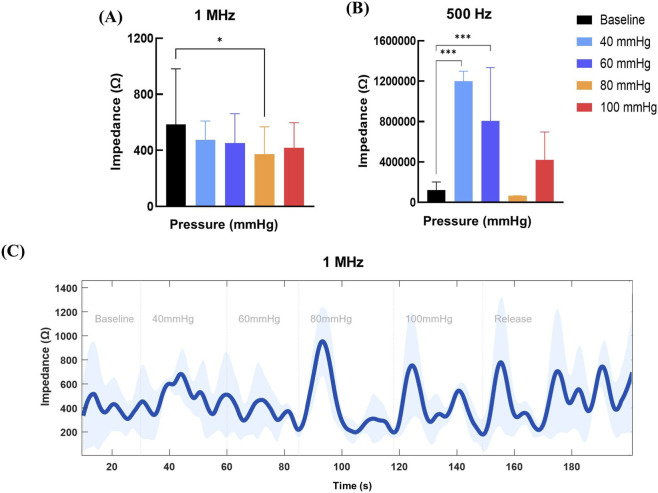
Effect of pressure on forearm bioimpedance (BioZ) measured with dry electrodes. **(A)** Mean BioZ (Ω) at High frequency (1 MHz). **(B)** Mean BioZ (Ω) at low Frequency (500 Hz). **(C)** Continuous BioZ (Ω) response at 1 MHz during incremental pressure application over time.

Forearm BioZ was first assessed at two frequencies 1 MHz (high) and 500 Hz (low) using dry surface electrodes under graded pressure (Figures 6A,B). At 1 MHz, significant differences were observed between baseline and high pressure (80 mmHg; p = 0.05; Figure 6A). At 500 Hz, BioZ demonstrated a nonlinear pressure response (Figure 6B), with impedance increasing sharply from baseline to low pressure and showing significant differences between baseline and both low and mid pressures (p = 0.001). The results indicated that 1 MHz exhibited a more responsive linear trend to pressure, whereas 500 Hz, although showing higher sensitivity, lacks a clear or consistent trend, making it less suitable for graded pressure detection.

Continuous monitoring at 1 MHz with incremental pressure steps showed a steady increase in BioZ, with values rising from a baseline of 400–600 Ω to approximately 1,000 Ω at mid pressure (Figure 6C). At the highest pressure, BioZ values dropped but did not return to baseline. Variability, indicated by the shaded region, increased progressively with pressures.

**FIGURE 7:**
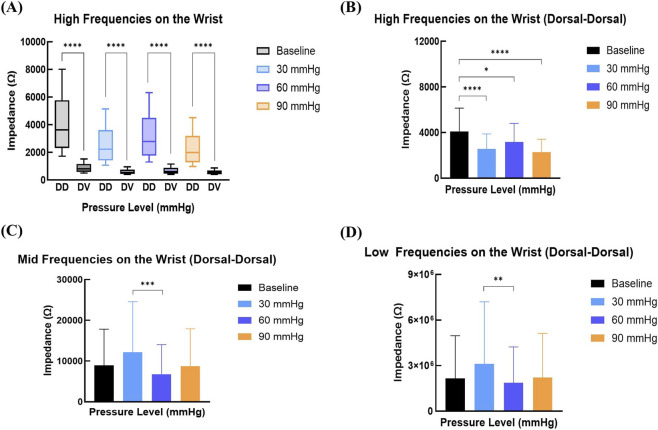
Effect of Pressure on Bioimpedance (BioZ) on the Wrist Using Dry Electrodes at High (>105 Hz), Mid (103 –105 Hz), and Low (<103 Hz) Frequencies Under Varying Pressure Levels (0, 30, 60, and 90 mmHg). **(A)** Box plot representing the mean BioZ change at different pressure levels across wrist locations (Dorsal-Volar and Dorsal-Dorsal). **(B)** High Frequency Wrist Mean BioZ (Dorsal-Dorsal). **(C)** Mid Frequency Wrist Mean BioZ (Dorsal-Dorsal). **(D)** Low Frequency Wrist Mean BioZ (Dorsal‐Dorsal).

To further examine how mechanical and electrical conditions affect BioZ in wearable contexts, measurements were performed at the wrist using dry electrodes (Figure 7A). BioZ responses were assessed across low, mid, and high frequency bands with two electrode configurations: Dorsal‐Dorsal (DD) and Dorsal‐Volar (DV). Incremental pressure was applied from 0 (baseline) to 90 mmHg, increasing in 30 mmHg steps. Significant differences were observed between the two electrode placements at all pressure levels (p = 0.001), with the DV configuration exhibiting lower median BioZ values and narrower IQR compared to DD.

High frequency measurements in the DD configuration showed a clear reduction in BioZ with increasing pressure (Figure 7B). Mean Bioz decreased from ∼4,000 Ω at baseline to ∼2,500 Ω at 30 mmHg (p = 0.001), with further reduction observed at 90 mmHg (p = 0.001) and minor reduction 60 mmHg (p = 0.05) compared to baseline. Mid-frequency responses (Figure 7C) demonstrated a moderate decrease in impedance with significant changes between 30 and 60 mmHg (p = 0.001). Low frequency measurements (Figure 7D) also decreased under pressure, with significant differences between 30 and 60 mmHg (p = 0.01), though the effect was less pronounced than at higher frequencies.

## 4 Discussion

This study investigated the relationship between localized BioZ, anthropometric measurements, and HGS in healthy young adults from the MENA region. The motivation lies in the need for population-specific data, as HGS values vary across ethnic and socioeconomic groups due to genetic, nutritional, and lifestyle differences (Arokiasamy et al., 2021; Wang et al., 2018). To our knowledge, this is the first study in the region to evaluate how segmental BioZ and limb circumference at the wrist and forearm relate to HGS, and to examine their integration with ML models. Our objectives were twofold (1): to evaluate how anthropometric and impedance features are associated with HGS; and (2) investigate how electrode site, frequency, and localized pressure influence impedance, thereby providing guidance for future wearable device design.

Compared to the wrist, the forearm yields more physiologically relevant signals for estimating grip strength (Kamaşak et al., 2023; Seethamma et al., 2019). This likely reflects the forearm’s direct involvement in grip mechanics where the flexor and extensor muscle groups extending from the forearm generate gripping force and stabilize the wrist (Seethamma et al., 2019). Computational modeling further supports this interpretation, demonstrating that electrode placement over muscle regions allows for wider and deeper current penetration and density distributions that would enhance the physiological relevance of BioZ recordings (Salkim and Wu, 2023). The observed negative correlation between forearm circumference and BioZ is consistent with this mechanism, as larger muscle cross-sectional area provides more conductive pathways for current, thereby reducing electrical resistance. Similar findings were reported in young adults, where forearm circumference, height, and body mass were identified as stronger predictors of HGS compared to wrist circumference (Kamaşak et al., 2023; Myles et al., 2025).

ML models integrating anthropometric and localized BioZ features improved HGS estimation compared to anthropometry alone. In baseline models, predictive performance was modest for both MLR and RF regression, indicating that global anthropometric descriptors capture limited variance in muscle strength (Figures 4A,B). Adding segmental forearm BioZ to baseline contributed complementary information alongside height and body mass, suggesting that localized electrical tissue properties provide a more comprehensive representation of grip strength beyond gross body size (Figure 5A). Notably, the strongest overall performance was achieved using a size-normalized forearm BioZ index that integrates both geometric (limb circumference) and electrical tissue properties (BioZ) (Figure 5C). This feature alone outperformed baseline anthropometric models in both linear and nonlinear frameworks, with RF regression achieving the lowest prediction error (RMSE_cv_ = 6.68 kg) (Table 2). While RF regression showed superior performance (Figure 5C), MLR performed comparably in simpler configurations (Figures 5A,B), indicating that performance gains were driven primarily by physiologically informed feature engineering rather than model complexity alone (Table 2). Although additional regression approaches (regularized and robust linear models) were explored during preliminary analyses, they did not provide performance improvements. We therefore focused on MLR and RF regression as representative linear and nonlinear frameworks that balance interpretability and overfitting risk in small-sample settings. This approach highlights the potential of localized, size-normalized BioZ features as informative inputs for ML-based estimation of muscle strength in healthy young adults, with the focus placed on physiologically informed feature design rather than model benchmarking.

While these findings point toward potential applications in wearable health monitoring, they should be interpreted with caution given the small pilot sample. Rather than claiming immediate translational impact, our results demonstrate proof-of-concept that localized, size-normalized forearm BioZ can enrich predictive models of grip strength being the most accurate and stable across all configurations. With validation in larger and diverse cohorts, such multimodal approaches may support non-invasive monitoring of musculoskeletal health, rehabilitation progress, and early signs of health decline.

In wearable applications, BioZ signals are influenced by dynamic factors such as movement, strap tightness, sweat, and skin-electrode contact variability (Cömert et al., 2013). Our findings highlight how frequency and pressure influence impedance responses. At low frequencies, current travels primarily through extracellular fluid pathways because the cell membranes act as capacitors. Under pressure, low frequency impedance responses were not consistent at the forearm, likely reflecting non-uniform displacement of extracellular fluid and blood. In contrast, high frequency currents penetrate cell membranes and engage both intra- and extracellular pathways. Impedance increased with pressure up to ∼80 mmHg, consistent with extracellular fluid reduction, cell compression, and changes in membrane capacitance (Dodde et al., 2012; Namkoong et al., 2024). Beyond this threshold, a plateau was observed, suggesting tissue compression limits had been reached (Namkoong et al., 2024). These findings indicate that high-frequency BioZ is more sensitive to localized fluid shifts or edema, whereas low-frequency signals provide less consistent information under varying pressure.

At the wrist, electrode configuration strongly influenced signal stability. While the DV placement produced more stable impedance values across pressure levels, it may not be practical for most wearable applications. In contrast, the DD placement exhibited higher baseline impedance and greater sensitivity to pressure-induced variation, suggesting a trade-off between robustness (DV) and responsiveness (DD) that should be carefully considered in wearable device design. In standard watch-style devices, the sensor contacts are limited to one side of the wrist. As a result, electrode placement must conform to a unilateral configuration such as DD, making it the more feasible option despite its higher sensitivity. The wrist’s thinner anatomy and lower muscle density likely explain the narrower impedance range compared with the forearm. Together, these results suggest that practical wearable devices should prioritize high-frequency BioZ measurements with DD electrode placement to maximize both feasibility and physiological relevance.

This study has limitations that should be acknowledged. The sample size was modest (n = 20), which constrains statistical power and limits the generalizability of ML models. In addition, the cohort consisted exclusively of healthy young adults from the MENA region; therefore, the findings may not extend to older populations or individuals with clinical conditions. All measurements were obtained under controlled laboratory conditions, which do not fully replicate the variability of real-world wearable use where factors such as movement, sweat, and strap displacement may degrade signal quality. Finally, ML models presented here should be viewed as exploratory. Larger, more diverse cohorts and external validation studies are required to establish robust predictive accuracy, enhance clinical relevance, and confirm the translational potential of this multimodal approach.

## 5 Conclusion

This study demonstrates that segmental forearm bioimpedance (BioZ), when combined with anthropometric measurements, provides a physiologically meaningful and complementary predictor of hand grip strength (HGS) in healthy young adults. By integrating BioZ with traditional anthropometry, we show that multimodal models particularly those using RF regression algorithms achieve higher predictive accuracy than linear models, underscoring the value of nonlinear approaches for capturing complex tissue function relationships. Importantly, our findings highlight the forearm as a superior measurement site compared to the wrist and reveal how factors such as frequency and applied pressure shape BioZ behavior, offering practical guidance for wearable sensor design. While preliminary, these results establish proof-of-concept that coupling BioZ with anthropometry can enrich predictive modeling of muscle strength and inform the development of next-generation wearable systems for continuous, non-invasive monitoring of musculoskeletal health, rehabilitation progress, and early indicators of functional decline.

## Data Availability

The raw data supporting the conclusions of this article will be made available by the authors, without undue reservation.
